# Liver Disease in Cystic Fibrosis: an Update

**DOI:** 10.5812/hepatmon.11215

**Published:** 2013-08-14

**Authors:** Giuseppe Fabio Parisi, Giovanna Di Dio, Chiara Franzonello, Alessia Gennaro, Novella Rotolo, Elena Lionetti, Salvatore Leonardi

**Affiliations:** 1 Department of Medical and Pediatric Science, Bronchopneumology and Cystic Fibrosis Unit, University of Catania, Catania, Italy

**Keywords:** Cystic Fibrosis, Liver Disease, Mutation

## Abstract

**Context:**

Cystic fibrosis (CF) is the most widespread autosomal recessive genetic disorder that limits life expectation amongst the Caucasian population. As the median survival has increased related to early multidisciplinary intervention, other manifestations of CF have emergedespecially for the broad spectrum of hepatobiliary involvement. The present study reviews the existing literature on liver disease in cystic fibrosis and describes the key issues for an adequate clinical evaluation and management of patients, with a focus on the pathogenetic, clinical and diagnostic-therapeutic aspects of liver disease in CF.

**Evidence Acquisition:**

A literature search of electronic databases was undertaken for relevant studies published from 1990 about liver disease in cystic fibrosis. The databases searched were: EMBASE, PubMed and Cochrane Library.

**Results:**

CF is due to mutations in the gene on chromosome 7 that encodes an amino acidic polypeptide named CFTR (cystic fibrosis transmembrane regulator). The hepatic manifestations include particular changes referring to the basic CFTR defect, iatrogenic lesions or consequences of the multisystem disease. Even though hepatobiliary disease is the most common non-pulmonary cause ofmortalityin CF (the third after pulmonary disease and transplant complications), only about the 33%ofCF patients presents clinically significant hepatobiliary disease.

**Conclusions:**

Liver disease will have a growing impact on survival and quality of life of cystic fibrosis patients because a longer life expectancy and for this it is important its early recognition and a correct clinical management aimed atdelaying the onset of complications. This review could represent an opportunity to encourage researchers to better investigate genotype-phenotype correlation associated with the development of cystic fibrosis liver disease, especially for non-CFTR genetic polymorphisms, and detect predisposed individuals. Therapeutic trials are needed to find strategies of fibrosis prevention and to avoid its progression prior to development its related complications.

## 1. Context

Cystic fibrosis (CF) is the most widespread autosomal recessive genetic disorder that limits life expectation amongst the Caucasian population. The approximate occurrence of CF is 1 in 3000 newborns (1). There is a great heterogeneity in clinical manifestations and the disease affects many organs but the most notable arelungs, reproductive tracts, pancreas,intestine and liver (2). CF is due to mutations in the gene on chromosome 7 that encodes an amino acidic polypeptide named CFTR (cystic fibrosis transmembrane regulator). CFTR is a protein acting as an anion channel and it is expressed on epithelial cells throughout the body (3). The alteration of chloride (Cl^−^) and sodium (Na^+^) transport, which results in water efflux abnormalities, is responsible for increasing the density of secretionsin different exocrine tissues (4). The most frequent mutation is p.Phe508del (∆F508) which represents about two thirds of all CFTR alleles in patients with CF, with an increased prevalence from Southeast Europe to Northwest (5). CFTR mutations can be divided into 6 different classes according to their predicted functional consequences for the CFTR protein (6, 7). Class I mutations alter the biosynthesis and results in the total or partial absence of the protein. Class II mutations, including p.Phe508del, lead to defective protein maturation and processing. Class III mutations disturb the regulation of chloride channel, leading to the formation of non-functional CFTR protein at the apical membrane. Class IV mutationslead to altered channel conductance. Class V mutations affect the stability of mRNA. Class VI mutations alter the stability of mature CFTR protein, leading to an unstable CFTR protein located on the apical membrane (8).

In general, class I, II, III and VI mutations are associated with pancreatic insufficiency, higher frequency of meconium ileus, premature mortality and are thus considered severe mutations. Class IV-V is usually associated with a mild disease. Class IV-V mutations are phenotypically dominant when occurring with class I, II, III, VI mutations (5).CF is no longer considered an exclusively pediatric disease as nowadays life expectation is over 35 years. As the median survival has increased related to early multidisciplinary intervention, other manifestations of CF have emergedespecially for the broad spectrum of hepatobiliary involvement. The hepatic manifestations include particular changes referring to thebasic CFTR defect, iatrogenic lesions or consequences of the multisystem disease (2). Even though hepatobiliary disease is the most common non-pulmonary cause ofmortalityin CF (the third after pulmonary disease and transplant complications), only about the 33%ofCF patients presents clinically significant hepatobiliary disease (9).

Lamireau et al. in a longitudinal study of prevalence in a widegroup of 241 people affected byCF demonstrated that cystic fibrosis liver disease (CFLD) occurs mainly in the first ten years oflife with a prevalence of 41% at 12 years of age but not later on. Cirrhosis occurred in 19 (7.8%) patients and liver transplantation was required in 5 patients (10). About 5 to 10% of every CF patients will be affected by multilobular cirrhosis withinthe first decade of life(11, 12). Liver failure is a late occurrence in CF patients, especially after the pediatric age and occasionally before it (13). The development and severity of CFLDhave not been connected to specific CFTR mutations, even if the involvement of liver and the progression of CFLD could have been delimited to patientspresenting a severe genotype. This highlights that the pathogenetic mechanism is produced by several factors. In addition to severe genotype, other risk factors of CFLD might include male gender, pancreatic insufficiency, severity of pulmonary disease and neonatal meconium ileus (14-16). Corbett et al. showed that patients diagnosed with CFLD suffer from worsened pulmonary conditions, poor growth and deterioration of nutritional status. They also reported that the early recognition of CF is very important because the delayed diagnosis facilitated the development of CFLD(15).

The present study reviews the existing literature on liver disease in cystic fibrosis and describes the key issues for an adequate clinical evaluation and management of patients with a focus on the pathogenetic, clinical and diagnostic-therapeutic aspects of CFLD.

## 2. Evidence Acquisition

A literature search of electronic databases was undertaken for relevant studies published from 1990 about liver disease in cystic fibrosis. The databases searched were: EMBASE, PubMed and Cochrane Library.

## 3. Results

### 3.1. Pathogenesis

The pathogenesis of CFLD is largely unknown. CFLD is due to the abnormal expression of CFTR in the apical surface of the biliary epithelium (17, 18). CFTR in biliary epithelium increases apical biliary chloride secretion primarily increasing bile acid independent bile flow (19). The abnormal activity or the absence of CFTR could decrease bile fluidity and alkalinity, causing accumulation and precipitation of hyperviscous biliary secretions in intrahepatic tree (12). Inspissated bile accumulates in the biliary ducts leading to cholangiocyte and hepatocyte injury, stimulating focal fibrosis (20). This has been hypothesized to lead to the accumulation of toxic bile acids in the liver, depletion of hepatic antioxidants, and liver cell injury. Repeated liver cell injury can activate hepatic stellate cells, which results in an increased production of the profibrogenic cytokine TGF-β, and can lead to hepatic fibrosis and in some cases cirrhosis (12, 21-23). Another theory of pathogenesis is that increased intestinal permeability in CF leads to absorption of pathogen associated molecular patterns that stimulate inflammation and fibrosis (24-28). It is unknown why only a subset of CF patients develops cirrhosis, while the majority of individuals with a similar CFTR defect do not develop cirrhosis [19]. Hepatobiliary manifestations occur almost exclusively in CF patients with severe class I, II, III and VI mutations (29) affecting CFTR synthesis, processing or regulation, but there are no clear phenotype relationships with specific CFTR mutations. In this regard, Wilshanski et al. screened 288 CF patients: 80 (28%) had liver disease. Of the 256 patients with pancreatic insufficiency, 80 (31%) had signs of liver disease; instead, of the 32 patients with pancreatic sufficiency, none had hepatic abnormalities. Genotype-phenotype correlation demonstrated the occurrence of liver disease in 56 (32%) of 173 patients bearing class I, II, III or VI mutations on both alleles and in 6 (38%) of 16 patients bearing at least one mutation associated with a variable genotype. These authors found a prevalence of liver disease increased with age but no correlation was found between liver disease and a history of meconium ileus, nutritional status or severity of lung disease (16).

Castaldo et al. in a multicentric study conducted in Southern Italy analyzed five sets ofCF siblings with different liver phenotype, 3 carrying genotype p.Phe508del/p.Arg553X (∆F508/R553X), 1 carrying genotype p.Phe508del/unknown, and 1 carrying genotype unknown/unknown. One sibling of each of the five sets was free of liver involvement, and the other had a severe liver expression, demonstrating that nutritional state, environmental factors, and therapy compliance are not involved in the liver expression of CF and modifier genes, inherited independently of the CFTR gene, might be important in the development of CFLD (30).

To determine the association between non-CFTR genetic polymorphisms and CFLD, Bartlett et al. in a two-stage case-control study enrolling patients with CF and severe liver disease with portal hypertension examined 9 functional variants in 5 genes previously studied in CFLD, including α1-antitrypsin (SERPINA1), angiotensin-converting enzyme (ACE), glutathione S-transferase (GSTP1), mannose-binding lectin 2 (MBL2), and transforming growth factor β1 (TGFB1). The initial study showed CFLD to be associated with the SERPINA1 Z allele and with TGFB1 codon 10 CC genotype. In the replication study, CFLD was associated with the SERPINA1 Z allele but not with TGFB1 codon 10. A combined analysis of the initial and replication studies by logistic regression showed CFLD to be associated with SERPINA1 Z allele. The authors concluded that patients who carry the Z allele are at greater risk (OR, ≈ 5) of developing severe liver disease with portal hypertension (31).

In 2012, Pereira et al. carried out a study to identify genes associated with hepatic injury and fibrosis in patients with CFLD, providing evidence for a transcriptional basis for the pathogenesis of CFLD. The authors demonstrated a different expression of several genes associated with hepatic fibrogenesis including, matrix metalloproteinases, collagens and chemokines in CFLD versus the control patients, particularly decreased expression in tissue remodelling genes including tissue inhibitor of metalloproteinase-1 (TIMP-1) and plasminogen activator inhibitor-1 (PAI-1, up to 25-fold) (32).These studies show a possible genetic predisposition, independent of the CFTR gene, in the pathogenesis of CFLD but further data are needed to add details about this issue.

### 3.2. Clinical Presentation

The wide spectrum of manifestations includes neonatal cholestasis, isolated elevated values of transaminases, hepatic steatosis, hepatic fibrosis, focal or multilobular cirrhosis with or without portal hypertension. The routine physical examination often shows an enlarged liver, even if the liver function tests could result normal (2, 12, 33). On clinical examination, the infants affected by neonatal cholestasis could appear with jaundice, which is observed also in patients with end-stage multilobular biliary cirrhosis(33). Steatosis is likely the most common hepatic finding in CF with a prevalence of 23–75% of CF patients in all age categories (34, 35). The underlyingCF secretory defect at the hepatobiliary level seems to not directly influence the pathogenesis of steatosis. It has not been demonstrated that steatosis progresses to cirrhosis and it is accepted considered as a relatively benign condition in CF. However, it is known that nonalcoholic steatohepatitis may cause cirrhosis in adults(36), therefore further studies are needed to add new data on this issue.Focal biliary fibrosis, characterized by variable portal fibrosis and eosinophilic concretions, is pathognomonic of CFLD and occurs in more than 20% of children and adolescents withCFLD (37). Due to the extension of the fibrogenic cascade, focal biliary cirrhosis evolves into multilobular cirrhosis and its related complications such as splenomegaly with hypersplenism, esophageal or gastric varices, ascites and encephalopathy (33).

Multilobular cirrhosis differs from focal biliary cirrhosis for the presence of multiple regenerative nodules and diffuses involvement of the liver. Clinically multilobular cirrhosis is detected by a hard nodular liver that may or may not be enlarged. Prior to the development of portal hypertension, there are often no other clinical features. Once portal hypertension is present, splenomegaly, upper gastrointestinal bleeding secondary to esophageal or gastric varices, or ascites may be the first suggestion of previously unsuspected cirrhosis (19). There is also another fatty infiltration pattern that is called pseudomasses. These typical structures can be described as hyperechoic areas 1-2 cm in diameter, which can be visualized as heterogenic liver parenchyma using imaging techniques(38, 39). Microgallbladder is reported in 5–45% of CF patients and 3–20% has gallbladder distension and evidence of gallbladder dysfunction. Older studies reported that gallstones develop in 3–25% of pediatric CF patients (34, 35). These stones are more commonly calcium bilirubinate stones (40). Morphological assessment could document abnormal cholangiographic findings referring to sclerosing cholangitis in children and adult patients with CFLD. The fibrogenic process stimulates an inflammatory cascade that involves biliary tract and causes proteinprecipitation and bile ducts compression, causing sclerosis cholangitis (38, 40).

Few case reports documented the occurence of hepatocellular carcinoma (HCC) in these patients. O' Donnel et al. reported a patient with documented CFLD for which he was under routine surveillance that presented with histologically proven HCC (41). Mckeon et al. documented the occurrence of HCC in a 32-years-old lady with a diagnosis of CFLD since early adolescence (42). Table 1 summarizes the hepatobiliary clinical findings in CF.The evolution of CFLD is usually slow and progressive: only about 10% of patients affected by CF and cirrhosis with portal hypertension progress to liver synthetic failure generally after pediatric age (14, 43, 44). 

**Table 1.** Hepatobiliary Clinical Findings in Cystic Fibrosis

	Frequency, %
**Focal biliary cirrhosis**	20-30
**Multilobular biliary cirrhosis**	5-15
**Portal hypertension**	2-5
**Microgallbladder**	15-45
**Cholelithiasis**	3-25
**Neonatal cholestasis**	< 10
**Sclerosing cholangitis**	rare
**Cholangiocarcinoma**	rare

### 3.3. Diagnosis

The identification of CFLD is based on regular clinical examination, liver function tests and imaging techniques (45). The occasional finding of hepatomegaly is the most common presentation of CFLD, often not associated with specific biochemical abnormalities (46). An intermittent elevation of transaminases or gammaglutamyltransferase (GGT) occurs in up to 50% of CF patients (47). However, an elevated GGT or alanine aminotransferase (ALT) have a low sensitivity of 50% and 52% and a specificity of 74% and 77% respectively (48). The presence of steatosis can be highlighted by an elevation of transaminases with normal concentrations of GGT and alkaline phosphatase. It has been shown that 50% of CD infants present this abnormality in the first year of life, but it normalizes within 3rd year of life(49).

Hepatobiliary system Ultrasonography (US) with Doppler is inexpensive and non-invasive and in addition is more sensitive for the diagnosis of CFLD thanbiochemical and clinical abnormalities (50, 51). This technique allows the high resolution study of the liver with assessment of size, hepatic texture, steatosis, gallbladder abnormalities and splenomegaly if associated (52). US can demonstrate multilobular nodularity indicative of cirrhosis, but is unreliable at detecting earlier stages of hepatic fibrosis. Steatosis and hepatic fibrosis may be indistinguishable. Children with normal hepatic US can have advanced fibrosis (13). Williams et al. reported in a nine-year review a lack of correlation between aspartate aminotransferase (AST) elevation and ultrasound findings in about 24% of cases (53).

According to Mueller-Abt et al., the exclusion of early CFLD is not reliably made with US because a normal US has only 33% of predictive value and sensitivity of 57% so the final diagnosis of liver fibrosis still requires biopsy. However a significantly abnormal US is a good predictor of advanced CFLD with a specificity of approximately 84% (54). Non-invasive liver elastography (Fibroscan) is a valuable tool to identify and quantify CFLD. In fact, Witter et al. in a case-control study performed fibroscan measurements in 66 CF patients and 59 controls and found a significant increaseof liver stiffness in patients with clinical CFLD(11.2kPa versus 5.1 kPa), biochemical CFLD (7.4kPa versus 5.4 kPa) or ultrasonographical CFLD (8.2 versus 4.3 kPa) (55). Portal hypertension can be investigated with US-Doppler. Vergesslich et al. investigated changes in portal venous hemodynamics in 32 patients with CF and found that CF patients had a diameter of the portal vein significantly increased than the control patients. Moreover, in CF patients over 12-year-old the mean flow volume of the portal vein showed a significant increase then the control patients. These authors suggested that the increase of diameter and flow volume of the portal vein is an adaptive mechanism in the pressure-volume relationship of the portal venous system in patients with CF (56).

Magnetic resonance (MR) imaging and MR cholangiopancreatography can be usedfor the assessment of pancreatic and hepatobiliary complications in CF. Liver abnormalities range from hepatomegaly and diffuse fatty infiltration to severe cirrhosis with fibrotic change, regenerative nodules, and portal hypertension. Alterations of biliary tree include cholelithiasis, stricturization, and narrowing or dilatation of the intra- and theextrahepatic bile ducts. Gallbladder abnormalities including microgallbladder are alsoeasily demonstrable. Other typical MR findings are pancreatic cysts and pancreatic duct abnormalities. MR cholangiopancreatography could help to assess the existence of biliary complications avoiding more invasive procedures and, together with MR imaging, may prove useful in the evaluation of patients with CF who possess abdominal symptoms that suggest hepatobiliary involvement (57).

**Figure 1.**
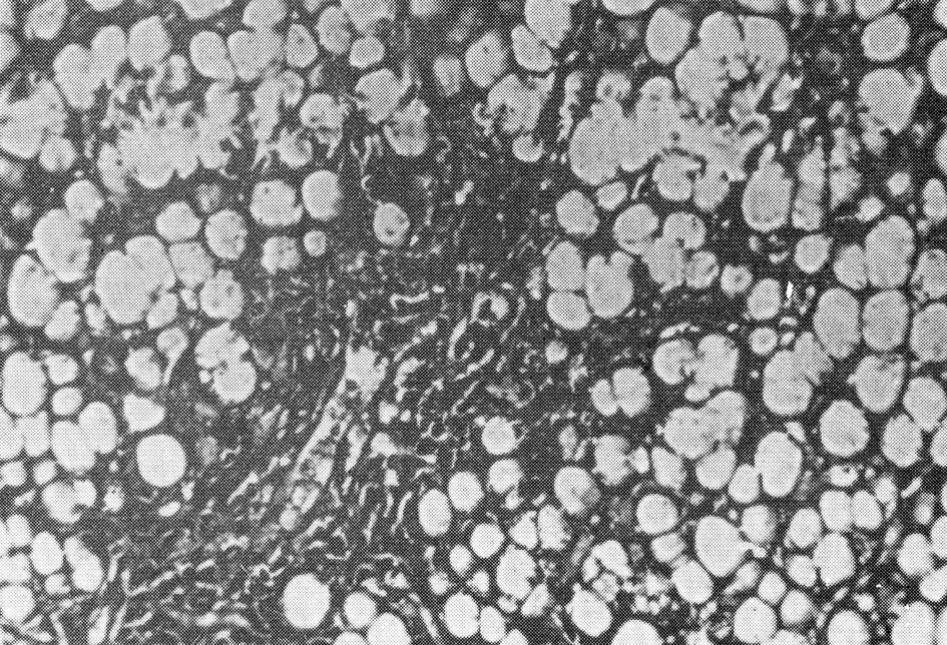
Portobiliary space enlarged for fibroblastic proliferation. Hepatic cells show signs of fatty degeneration with the total disappearance of nuclei.

**Figure 2.**
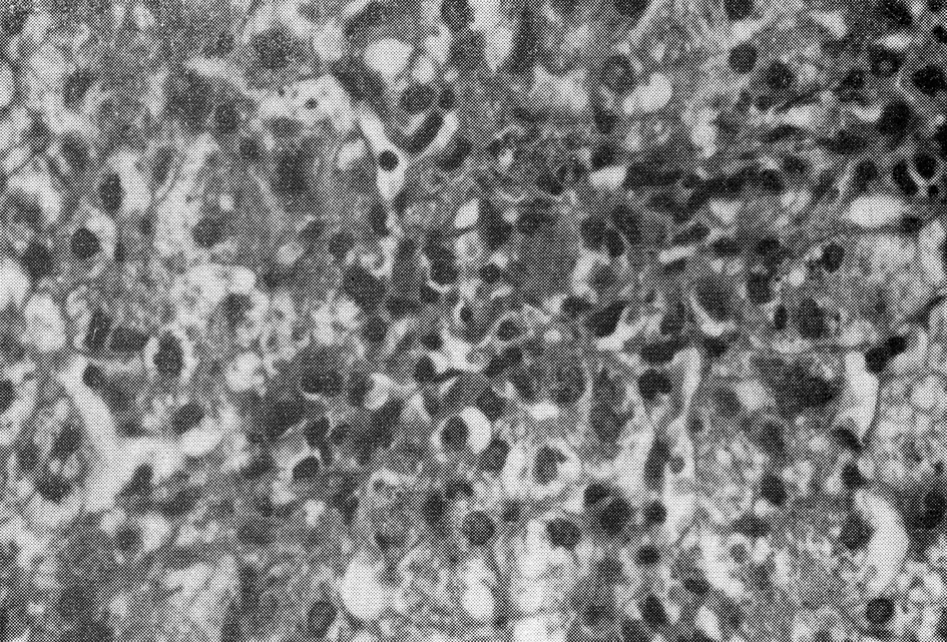
Portobiliary space enlarged for parvocellular infiltration and fibroblastic proliferation. Presence of intrahepatic biliary pigment and biliary thrombi.Presence of signs of extramedullary hematopoiesis.

Computed tomography (CT), differently from MR, exposes the patient to x-rays and does not allow imaging of the biliary tree. The liver may have decreased attenuation (creating a vascular prominence) or appear normal on CT. If portal hypertension is present, splenomegaly, portosystemic shunts, portal vein enlargement, hepatofugal flow and ascites can be observed (58-60). Liver biopsy is an invasive procedure associated with significant morbidity and mortality. Nevertheless,biopsy could provide several pieces of integral information on the nature of the lesion (focal biliary cirrhosis or steatosis), on the extent of portal fibrosis, on response to treatmentor worsening of fibrotic grade and could establish the need for liver transplantation (Figures 1 and 2) (46). 

Several studies have suggested the use of serum markers of hepatic fibrogenesis for early detection of CFLD, such as collagen type IV (CL-IV), tissue inhibitor of metalloproteinase (TIMP-1), prolyl hydroxylase (hPH) and glutathione S-transferase(22, 61). Pereira et al. showed that serum CL-IV, TIMP-1 and hPH and have raised levels in patients with CFLD. Moreover, serum TIMP-1 and hPH levels are increased in early hepatic fibrogenesis, returning to control levels with progression to advanced fibrosis. Thus, they could be useful in the early detection of liver disease in CF, particularly when used in combination (22). Wyatt et al. analyzed serum hyaluronic acid (HA) concentrations in patients with CFLD. HA is a glycosaminoglican rapidly metabolized by the liver. Increased serum HA levels could be due to an impairment of endothelial cell function caused by liver disease or an enhanced synthesis in the liver during hepatic fibrogenesis. These authors measured serum HA concentrations in a total of 74 patients with CF and 293 controls. The CF patients which had no evidence of CFLD had serum HA levels similar to controls; instead, in CF patients with a clinical and US diagnosis of CFLD serum HA levels was significantly increased. In CF patients with an abnormal liver US scan alone, although to a lesser extent, serum HA concentrations were also significantly increased.These authors proved that serum HA levels are increased in patients with clinical or US evidence of CFLD, being higher in those with more advanced hepatic damage and longitudinal measurement of HA levels may prove a useful marker for the development of significant liver damage in CF patients (62).

In 2013, two novel and powerful markers of CFLD were identified, tissue inhibitor of metalloproteinase-4 (TIMP-4) and Endoglin (homodimer of 180 kDA located on cell surfaces and part of TGF-β receptor complex). In ELISA quantifications, TIMP-4 and Endoglin were significantly up-regulated in patients with CFLD and their expression correlated with hepatic staging (63). In summary, according to Debray et al., the diagnostic criteria of CFLD are (Table 2): 

• Pathological changes on physical examination: hepatomegaly and/or splenomegaly, confirmed by US.

• Pathological levels of liver function tests (increase of transaminases AST and ALT and GGT levels above the upper normal limits at least at 3 consecutive determinations over 12 months after excluding other causes of liver disease).

• Ultrasonographic aspects of liver disease (irregular margins, increased and/or heterogeneous echogenicity, nodularity) or portal hypertension (splenomegaly, ascites, large collateral veins, spontaneous splenorenal anastomosis, increased thickness of the lesser omentum) or biliary abnormalities (bile duct dilatation).

• A liver biopsy may be indicated if there is a diagnostic doubt.

The presence of CFLD should be considered if at least two of these variables are present.

Therefore, annual screening of cystic fibrosis patients is recommended with:

• Abdominal examination by a gastroenterologist.

• Biochemical evaluation (AST, ALT, GGT, ALP, Prothrombin time, platelets).

• Abdominal US with CT or MR imaging if concern exists about the nature of liver lesions or biliary tract involvement (such as sclerosing cholangitis).

**Table 2.** Diagnostic Criteria for Liver Disease According to Debray et al. (45)

At Least Two of the Following Variables	
**1**	Abnormal physical examination
**a**	hepatomegaly confirmed by US ^a^
**b**	splenomegaly confirmed by US
**2**	Abnormalities of liver function tests
**3**	US evidence of liver involvement

^a^ Abbreviation: US, Ultrasound

Annual follow-up by hepatologists in all patients with CFLD is important to evaluate cirrhosis progression and to avoid the development of portal hypertension and its complications (esophageal varices, hepatopulmonary syndrome pulmonary arterial hypertension, thrombocytopenia and leukopenia, liver failure) (45).

### 3.4. Treatment

Currently, the only therapy that may prevent or avoid progression of CFLD is Ursodeoxycholic Acid (UDCA) that increases bile flow, replaces potentially toxic bile acids, acts as a cytoprotective agent, and possibly stimulates bicarbonate secretion in the biliary tract (23). Smith et al. demonstrated a significantly greater proportion of endogenous biliary UDCA in CF patients without liver disease compared to subjects with CFLD, confirming the protective role of UDCA(64). Generally, the recommended dose is 20 mg/kg/day of UDCA in two or three divided doses when there are US alterations and/or persistent increased liver damage tests. It is well tolerated and itsmain side effect is diarrhoea, in which case reducing the dose should prove sufficient (65).

In a retrospective study, Siano et al. demonstrated a higher prevalence of CFLD in patients with meconium ileus treated with UDCA at the onset of CFLD than those treated early with UDCA, before the onset of CLFD. This data suggests that an early treatment with UDCA may have some benefit also in patients at risk of developing CFLD such as those with meconium ileus (66).Liver transplantation can be considered for CFLD patients. Unfortunately, several questions about liver transplantation remain unsolved because, although CFLD were identified in a large number of studies, only a small number of patients were treated with liver transplantation (67-70). Indications for liver transplantation in CF liver disease are reported in table 3 (45).

**Table 3.** Indications for Liver Transplantation

	Description
**1**	gradual hepatic impairment unresponsive to standard treatment
**2**	presence of jaundice or ascites
**3**	presence of variceal bleeding
**4**	development of portopulmonary or hepatopulmonary syndromes
**5**	severe malnutrition, unresponsive to intensive nutritional support
**6**	impaired quality of life secondary to liver disease
**7**	impaired pulmonary function

During long-term follow-up survival rates of recipients with CF are lower than recipients with other indication for liver transplantation (71). In the retrospective study of Molmenti et al. the mean patient and graft survival of those who died after transplantation was 2.35 years; all were below the 5th percentile for height and weight at the time of liver transplantation and all had a history of meconium ileus and preoperative need of pancreatic enzymes (72). In CF patients the association of malabsorption and impairednutritional condition, worsening pulmonary status, and infection could have a negative prognostic value and could increase morbidity and mortality after liver transplantation. Therefore, liver transplantation is restricted to patients with intractable complications of portal hypertension and/or end-stage liver failure in good nutritional state (71, 72). Combined lung and liver transplantation has been performed but the outcome has not been satisfactory, with survival rates at one and five years of 69% and 49% respectively (73, 74).

## 4. Conclusions

Liver disease will have a growing impact on survival and quality of life of cystic fibrosis patients because a longer life expectancy and for this it is important its early recognition and a correct clinical management aimed atdelaying the onset of complications.This review could represent an opportunity to encourage researchers to better investigate genotype-phenotype correlation associated with the development of CFLD, especially for non-CFTR genetic polymorphisms, and detect predisposed individuals. Therapeutic trials are needed to find strategies of fibrosis prevention and to avoid its progression prior to development its related complications.
